# Therapeutic and Prognostic Implications of Immune-Related Adverse Events in Advanced Non-Small-Cell Lung Cancer

**DOI:** 10.3389/fonc.2021.703893

**Published:** 2021-06-29

**Authors:** Lea Daniello, Mariam Elshiaty, Farastuk Bozorgmehr, Jonas Kuon, Daniel Kazdal, Hannah Schindler, Rajiv Shah, Anna-Lena Volckmar, Fabienne Lusky, Leonore Diekmann, Stephan Liersch, Martin Faehling, Thomas Muley, Mark Kriegsmann, Karolina Benesova, Albrecht Stenzinger, Michael Thomas, Petros Christopoulos

**Affiliations:** ^1^ Department of Thoracic Oncology, Thoraxklinik and National Center for Tumor Diseases (NCT) at Heidelberg University Hospital, Heidelberg, Germany; ^2^ Translational Lung Research Center Heidelberg (TLRC-H), Member of the German Center of Lung Research (DZL), Heidelberg, Germany; ^3^ Institute of Pathology Heidelberg, Heidelberg, Germany; ^4^ Department of Internal Medicine V, Hematology, Oncology und Rheumatology, Heidelberg University Hospital, Heidelberg, Germany; ^5^ Department of Pharmacy, Thoraxklinik at Heidelberg University Hospital, Heidelberg, Germany; ^6^ Department of Cardiology, Angiology and Pneumology, Esslingen Hospital, Esslingen, Germany; ^7^ Translational Research Unit, Thoraxklinik at Heidelberg University Hospital, Heidelberg, Germany

**Keywords:** immune-related adverse events, immunotherapy, immune-checkpoint inhibitors, treatment interruption, prognosis, lethality

## Abstract

**Introduction:**

PD-(L)1 inhibitors have improved prognosis of non-small-cell lung cancer (NSCLC), but can also cause immune-related adverse events (irAEs) that complicate management.

**Methods:**

We analyzed NSCLC patients receiving PD-(L)1 inhibitors from 2012 to 2020 in a German academic center.

**Results:**

IrAE showed comparable frequencies in stage IV (198/894 or 22%) *vs.* III (14/45 or 31%, p = 0.15), after anti-PD-(L)1 monotherapy *vs.* chemoimmunotherapy (139/483 *vs.* 58/213, p = 0.75), and across treatment lines. In stage IV, irAE occurred after 3.1 months in median, affected multiple organs (median 2) in 27/894 patients and were associated with PD-L1 positivity (25 *vs.* 14%, p = 0.003), lower neutrophil-to-lymphocyte ratios (29 *vs.* 17%, p < 0.001 for NLR dichotomized at 5), better ECOG status (26 *vs.* 18% for 0 *vs.* 1, p = 0.004), but not related to age, sex, smoking and palliative radiotherapy. Two hundred thirty two irAEs occurred mostly in endocrine glands (4.9%), lungs (4.4%), the musculoskeletal system (4.2%), colon (4.1%), liver (3.7%), and skin (2.6%), while pneumonitis was most frequent with durvalumab following definitive chemoradiation (16% or 7/45, p < 0.01). IrAE severity was grade 1 in 11%, 2 in 41%, 3 in 36%, and 4 in 11% events, while two were lethal (<1%, myocarditis and pneumonitis). Therapy was suspended in 72%, while steroids were initiated in 66% and complemented by other immunosuppressants in 6%, with longest treatment duration for rheumatic events (mean >3 months), and average cumulative prednisone doses >700 mg for all organs, except for skin. Patients developing irAE had longer progression-free (PFS) and overall survival (OS) in multivariable 12/14-week landmark analyses including ECOG status, treatment line, treatment type, PD-L1 TPS, and NLR (median PFS 17 *vs.* 10 months, HR = 0.68, p = 0.009; median OS 37 *vs.* 15 months, HR = 0.40, p < 0.001), regardless of grade. OS was longest with skin (95% at 2 years) and shortest with pneumonitis, hepatitis, neurologic, and cardiologic irAE (38, 37, 28, and 0% at 2 years, p < 0.001).

**Conclusions:**

Approximately one-fourth of immunotherapy-treated NSCLC patients develop irAEs, most of which necessitate treatment suspension and steroids. Despite more frequent occurrence with PD-L1 positive tumors, lower NLR, and better ECOG PS, irAEs are independently associated with longer survival, especially when affecting the skin. Lethality is below 1%.

## Introduction

Inhibitors of immune checkpoints (ICIs), such as the cytotoxic T-lymphocyte-associated protein 4 (CTLA-4), programmed cell death protein-1 (PD-1), and its ligand programmed death-ligand 1 (PD-L1), are increasingly used for the treatment of metastatic cancers (1). These drugs block inhibitory effects of neoplastic on immune cells, to potentiate immunologic tumor control (2). Nivolumab, pembrolizumab, ipilimumab, and atezolizumab have improved progression-free (PFS) and overall survival (OS) of patients with metastatic non-small-cell lung cancer (NSCLC) in randomized phase 3 trials and currently represent the standard first-line treatment alone or in combination with chemotherapy for most cases (3, 4).

Besides the high antitumor efficacy of ICIs, as exemplified by an unprecedented 5-year survival rate of 32% for stage IV patients with PD-L1 high-expressing NSCLC receiving first-line pembrolizumab monotherapy (5), these drugs can also alter the physiology of immune responses, leading to toxicity collectively described as “immune-related adverse events” (irAEs), which can affect diverse organs and complicate patient management (6). The severity grading for irAEs relies on the National Cancer Institute common toxicity criteria for adverse events (NCI CTCAE) version 5 (7). Grade ≥3 toxicities, especially, can be even life threatening and require special monitoring and therapeutic maneuvers, including dose reductions, treatment interruption, and/or high-dose steroids (8).

With increasing use of ICIs as treatment for NSCLC, precise characterization of predisposing factors, manifestations, management, outcome, and impact on overall prognosis becomes more important for irAEs, because this knowledge could become a valuable aid for decision-making in daily clinical practice. This retrospective study utilizes a large, single-institution cohort to address these issues under real-world conditions.

## Patients and Methods

### Study Population and Data Collection

This study was approved by the ethics committee of Heidelberg University (S-296/2016) and included all advanced NSCLC patients treated with PD-(L)1 inhibitors in the Thoraxklinik Heidelberg between October 2012 and June 2020. Patients that received other immunotherapies, in particular CTLA-4 inhibitors, were excluded from this analysis.

**Figure 1:**
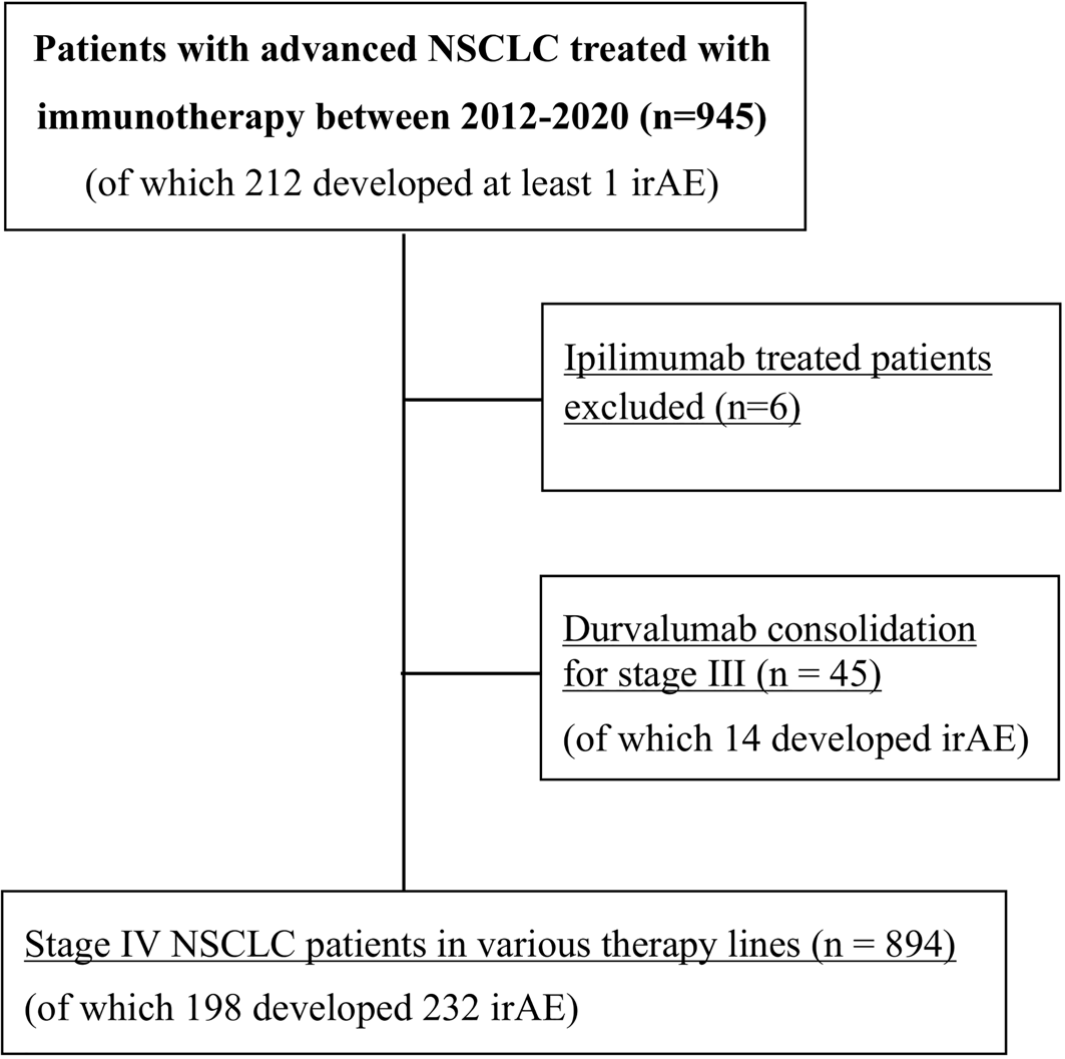
Flowchart of study patients. NSCLC, non-small-cell lung cancer; irAE, immune-related adverse events.

Diagnosis of NSCLC was performed in the Institute of Pathology Heidelberg using tissue specimens according to the criteria of the current WHO classification (2015) for lung cancer, as described previously (9, 10). Clinical data and laboratory results were collected by a systematic review of patient records. The following clinical data were extracted: demographic, baseline clinical and tumor characteristics, including ECOG performance status (PS), smoking status, PD-L1 tumor proportion score (TPS), laboratory results, systemic and local anticancer treatments, date of progression, date of the last follow-up, and date of death. The neutrophile-to-lymphocyte ratio (NLR) was dichotomized at the bibliographical cut-off of 5, which corresponds to the median value for untreated patients (11, 12). PD-L1 TPS was assessed using the clone SP263 (Ventana/Roche, Mannheim, Germany) and trichotomized for analysis as <1, 1–49, and ≥50%. For calculation of PFS, the progression date under immunotherapy was verified by the investigators with review of radiologic images, *i.e.* chest/abdomen CT and brain MRI-based restaging every 6–12 weeks, without formal RECIST reevaluation, as several studies have demonstrated very good agreement between real-world and RECIST-based assessments (13, 14). Patients with irAEs were diagnosed based on clinicolaboratory criteria and treated according to the current guidelines (7, 15). Diagnosis of pneumonitis was based on high-resolution CT (HRCT), considering that bioptic confirmation is generally not required for subsequent patient management (15). For patients with irAE additional data were collected about severity, management, outcome, and impact of irAEs on anticancer treatment. The subset of stage III patients who received durvalumab as consolidation after chemoradiation was analyzed separately (**Figure 1**).

### Detailed Characterization of irAEs

Patients who developed irAEs were analyzed further in more detail regarding affected organs and severity grade, time of onset, treatment with steroids—including start date, dose, duration, whether anticancer therapy had to be interrupted or terminated—or other immunosupressants, and if radiotherapy had been given in the past. Rheumatic irAEs were diagnosed in consultation with an experienced rheumatologist (KB) in order to differentiate them from non-autoimmune joint disease (*e.g.* osteoarthritis) (16, 17).

### Statistical Methods

Categorical data were analyzed using the chi-square test, including “goodness-of-fit” tests for the observed frequencies against the even distribution, when applicable. Numerical data were compared across two groups using an unpaired t-test and across three or more groups using one-way ANOVA with the Dunnett’s *post-hoc* test with correction for multiple testing. Survival data were analyzed according to Kaplan–Meier and compared between groups with the logrank test. The association of irAE and other variables with survival was analyzed using Cox regression. Immortal time bias was controlled through two landmark analyses, at 12 and 14 weeks, which included only cases surviving beyond the respective landmark, as well as by a time-dependent Cox regression, in which the occurrence of irAEs was considered as time-dependent covariate. Statistical calculations were performed with SPSS version 27 (IBM Corp., Armonk, NY, USA), and plots were generated with SPSS and Microsoft Excel 365 (Redmond, WA, USA). P-values <0.05 were considered as statistically significant.

## Results

### Study Population and Overview of irAEs

**Figure 2:**
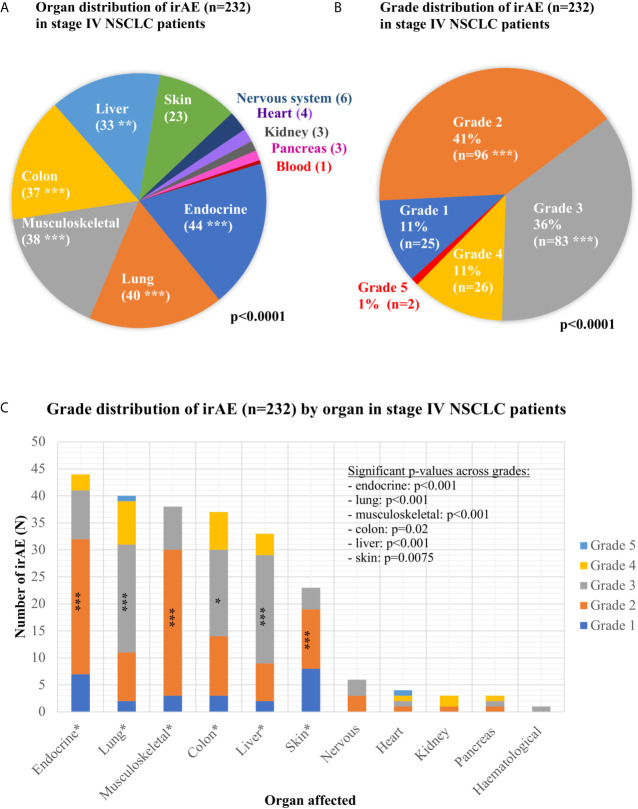
Organ and grade distribution of immune-related adverse events in immunotherapy-treated non-small-cell lung cancer patients. **(A)** Organ distribution of the 232 immune-related adverse events (irAEs) observed in stage IV non-small-cell lung cancer (NSCLC) patients (p < 0.0001 with a chi-square test across the various affected organs; detailed results are shown in **Table 2**; ***p < 0.001, **p < 0.01, *p < 0.05). **(B)** Grade distribution of the 232 irAEs observed in stage IV NSCLC patients (p < 0.0001 with a chi-square test across grades, grades with significantly increased frequency are marked with asterisks). **(C)** Grade distribution of the irAEs observed in each organ for stage IV NSCLC patients. For each organ, the p-value was calculated with a chi-square test of the observed frequencies for each grade against the even distribution (endocrinological: 44 irAEs overall, grade 1:7, grade 2:25, grade 3:9, grade 4:3, p < 0.001; lungs: 40 irAEs overall, grade 1:2, grade 2:9, grade 3:20, grade 4:8, grade 5:1, p < 0.001; musculoskeletal system: 38 irAEs overall, grade 1:3, grade 2:27, grade 3:8, grade 4:0, p < 0.001; colon: 37 irAE overall, grade 1:3, grade 2:11, grade 3:16, grade 4:7, p = 0.02; hepatitis: 33 irAEs overall, grade 1:2, grade 2:7, grade 3:20, grade 4:4, p < 0.001; skin: 23 irAEs overall, grade 1:8, grade 2:11, grade 3:4, grade 4:0, p = 0.0075; nervous system: six irAEs overall, grade 1:0, grade 2:3, grade 3:3, grade 4:0, p = 0.06; heart: four irAEs overall, grade 1:0, grade 2:1, grade 3:1, grade 4:1, grade 5:1, p = 0.26; kidneys: three irAEs overall, grade 1:0, grade 2:1, grade 3:0, grade 4:2, p = 0.30; pancreas: three irAEs overall, grade 1:0, grade 2:1, grade 3:1, grade 4:0, p = 0.80; hematological: one irAE overall, grade 3).

Overall, 939 consecutive patients were included in the study, of which 894 were treated with ICI monotherapy (70%) or chemoimmunotherapy (30%) for metastatic disease as shown in **Table 1**, while 45 patients received durvalumab after chemoradiotherapy of locally advanced tumors (**Figure 1** and **Supplementary Table 1**). Mean age was 65 years, with a predominance of male (60%) and smokers (92%) showing mostly an ECOG PS of 0–1 (98%). IrAEs showed comparable overall frequencies in stage IV *vs.* III (22 *vs.* 31%, p = 0.15), after ICI monotherapy *vs.* chemoimmunotherapy (22 *vs.* 21%, p = 0.75), across treatment lines (21–26% in the first *vs.* 20–33% in subsequent lines, p = 0.08–0.68), and across different ICIs (p = 0.16–0.74), with a trend for lower frequency for PD-L1 compared to PD-1 inhibitor monotherapy (13 *vs.* 23%, p = 0.053, **Table 1**). Among stage IV patients, 232 irAEs were documented, with involvement of multiple organs (two in median) in 14% (27/198) of patients. Most frequently affected were the endocrine glands (in 4.9% of patients, or 44/894), lungs (4.4%), musculoskeletal system (4.2%), colon (4.1%), and liver (3.7%), followed by the skin (2.6%), nervous system (0.7%), heart (0.4%), kidney (0.3%), pancreas (0.3%), and blood (0.1%, p < 0.001 across organs, **Figure 2A** and **Table 2**). CTCAE severity was grade 1 in 11% (25/232), grade 2 in 41%, grade 3 in 36%, and grade 4 in 11% (p < 0.001 across grades, **Figure 2B**), while two events were lethal (2/939 = 0.2%, one instance of myocarditis, and one instance of pneumonitis). The percentages of patients with at least one grade 3–4 irAE was comparable between stage IV (11% or 97/894) and stage III (18% or 8/45, p = 0.14, **Supplementary Table 2**) patients. In stage IV, the severity distribution was skewed for several organs, with predominance of grade 2 irAE for the skin (p = 0.0075), endocrine (p < 0.001), and musculoskeletal systems (p < 0.001), while grade 3 was more frequent for pneumonitis (p < 0.001), colitis (p = 0.02), and hepatitis (p < 0.001, **Figure 2C**). Besides, in stage III patients after definitive chemoradiation, grade ≥3 pneumonitis predominated (6/14, **Supplementary Table 2**).

**Table 1:** Characteristics of stage IV NSCLC patients.

All study patients (N = 894)		No irAE (N = 696)	With irAE (N = 198)	p-value
Age, median; IQR		65;12	65;12	0.57
Sex, male/female		419/272	117/81	0.67
Never/light-smokers (<10 py)		70/650	20/186	0.60
Pack–years, mean (SE)		38 (1.0)	40 (2.0)	0.34
**ECOG PS, median (IQR)**		1 (1)	0 (1)	0.016
PD-L1 TPS ≥1/<1%, n (%)		489/140	159 (24)/22 (14)	0.003
PD-L1 TPS, mean (SE)		34.3 (1.5)	42.6 (2.8)	0.008
NLR ≥5/<5, n (%)		444/233	93 (17)/98 (29)	<0.001
NLR, mean (SE)		9.0 (0.3)	7.0 (0.7)	0.005
ICI-monotherapy, n (% of ICI-monotherapy)		483	139 (22)	0.75
Chemo-IO, n (% of Chemo-IO)		213	58 (21)	
ICI-monotherapy, 1L, n (% of first line)		159	56 (26)	0.08
ICI-monotherapy, lines 2–8, (% of later lines)		324	83 (20)	
CHT-IO, 1L, n (% of first line)		198	53 (21)	0.68
CHT-IO, lines 2–8, (% of later lines)		15	5 (25)	
ICI drug, 1L, n (% of drug)	Pembrolizumab	118	48 (29)	0.17
	Nivolumab	35	9 (20)	
	Atezolizumab	6	0 (0)	
ICI drug, lines 2–8, n (% of drug)	Nivolumab	213	54 (20)	0.16
	Pembrolizumab	62	20 (24)	
	Atezolizumab	46	8 (15)	
	Durvalumab	3	0 (0)	
ICI type, across lines, n (%)	PD-1 inhibitor	428	131 (23)	0.053
	PD-L1 inhibitor	55	8 (13)	
CHT-IO, 1L, n (% of drug)	CHT + pembrolizumab	189	49 (21)	0.67
	CHT + atezolizumab	4	2 (33)	
	CHT + durvalumab	5	2 (29)	
CHT-IO, lines 2–8, n (% of drug)	CHT + pembrolizumab	4	2 (33)	0.74
	CHT + atezolizumab	9	2 (18)	
	CHT + durvalumab	2	1 (33)	
CHT-IO, across lines, n (%)	CHT + PD-1 inhibitor	193	51 (21)	0.55
	CHT + PD-1 inhibitor	20	7 (26)	
Any radiotherapy		246/701	63/198	0.37
Thoracic radiotherapy (with respect to pneumonitis)		110/860	6/40	0.68

ECOG PS, ECOG performance status; ICI, immune checkpoint inhibitor; IO, immunotherapy; 1L, first line; NLR, neutrophil-to-lymphocyte ratio. All bold values of the table show a significance of p< 0.05.

**Table 2:** Severity, onset, and management of immune-related adverse events in stage IV non-small-cell lung cancer patients.

	IrAE grade and impact on ICI administration					Steroid treatment			
	Any grade, % (n) of all patients	Time to onset(days)	G≥3, % (n) of each organ	ICI suspension, % (n) of each organ	ICI termination, % (n) of each organ	Steroids,% (n) of each organ	Initial daily dose, ^1^ mean (SD)	Average daily dose, ^1^ mean (SD)	Duration, days (SD)
**Affected organ**									
Endocrine	4.9 (44***)	132	27 (12)	43 (19)	16 (7)	27 (12)	18 (33)	12 (26)	31 (107)
Lungs	4.4 (40***)	105	73 (29***)	95 (38***)	80 (32***)	93 (37***)	75 (41)***	53 (60)***	41 (36)
Musculoskeletal	4.3 (38***)	246*	21 (8)	58 (22)	47 (18)	74 (28***)	33 (52)	20 (36)	128 (202)***
Colon	4.1 (37***)	168	62 (23*)	84 (31***)	65 (24***)	81 (30***)	62 (44)*	40 (34)*	44 (42)
Liver	3.7 (33**)	67	73 (24**)	94 (31***)	82 (27***)	82 (27***)	87 (92)***	47 (37)***	33 (27)
Skin	2.6 (23)	182	17 (5)	52(12)	35 (8)	35 (8)	21 (36)	9 (15)	23 (55)
Nervous system	0.7 (6)	52	67 (4)	100 (6)	67(4)	67 (4)	111 (196)*	35 (39)	21 (23)
Heart	0.4 (4)	75	100 (4)	100(4)	75 (3)	50 (2)	135 (244)**	36 (45)	39 (61)
Kidney	0.3 (3)	669***	67 (2)	100 (3)	100 (3)	100 (3)	67 (29)	42 (12)	86 (97)
Pancreas	0.3 (3)	311	67 (2)	67 (2)	67 (2)	67 (2)	52 (50)	23 (21)	53 (65)
Blood	0.1 (1)	838	100 (1)	100 (1)	100 (1)	100 (1)	100 (n/a)	57 (na)	20 (na)
**p-value**	<0.001	<0.001	<0.001	0.003	<0.001	<0.001	<0.001	<0.001	0.002

IrAEs of various organs are listed in order of decreasing frequency according to **Figure 2C** (n = 894 stage IV patients). Statistical comparisons to detect increased values across organs were performed using either a chi-square test against the even distribution (frequency of irAEs with any grade or grade ≥3, rates of ICI suspension (that is interruption or termination), or termination, rate of steroid treatment, or one-way ANOVA (time to onset, dose and duration of steroids), followed by the Dunnett’s post-hoc test with endocrine irAE as reference. Statistically significant results are highlighted in bold.

G, grade 3; irAE, immune related adverse events; ICI, immunotherapy; SD, standard deviation; n/a, not applicable; ***p < 0.001; **p < 0.01; *p < 0.05.

^1^in mg prednisone.

### Clinical Characteristics Associated With Occurrence of irAEs

Most patient characteristics, like age, sex, and smoking status, were balanced between patients with or without irAE (**Table 1**). At the same time, irAE occurrence was significantly associated with PD-L1 positivity, *i.e.* TPS ≥1% (25 *vs.* 14%, p = 0.003; mean PD-L1 TPS 43 *vs.* 34% for patients with *vs.* without irAE, p = 0.008), a lower baseline NLR <5 (29 *vs.* 17% for patients with NLR ≥5, p < 0.001; mean NLR 7.0 *vs*. 9.0, p = 0.005), and a better ECOG PS (26 *vs.* 18% for PS 0 *vs.* 1, p = 0.004). In stage IV patients, there was no significant relationship between administration of palliative radiotherapy to any organ and development of any irAE (p = 0.37), or between prior palliative thoracic radiotherapy and development of pneumonitis p = 0.68, **Table 1**). However, the frequency of pneumonitis was significantly higher in stage III patients receiving durvalumab after curative-intent radiotherapy compared to stage IV patients (7/45 = 16% *vs.* 40/894 = 4%, respectively, p = 0.0009). Similarly, the relative frequency of pneumonitis among the observed irAE was significantly higher for stage III compared to stage IV patients (7/14 = 50% *vs.* 40/232 = 17%, respectively, p = 0.0025, **Table 2** and **Supplementary Table 2**). Median time-to-onset of irAE from ICI start was 3.1 months (92 days), with significantly later onset for musculoskeletal (246 days, p = 0.046) and renal events (669 days, p < 0.001, **Table 2**).

### Management of irAEs

The majority of irAE interfered with further administration of ICI therapy, leading to suspension in 72% (168/232), and termination in 55% (128/232) of cases, respectively (**Table 2**). This affected practically all patients with grade 3–4 events (98 and 100%, respectively), but ICI therapy was also permanently discontinued for 21% of patients with grade 1 (n = 4), and 51% of patients with grade 2 events (n = 35). There were considerable differences depending on the affected organ, with significantly more pneumonitis (95%), colitis (84%), and hepatitis (94%) irAEs leading to suspension (p < 0.001, **Table 2**). For irAEs of other vital organs, *i.e.* nervous system, heart, kidneys, and blood, the suspension rate was also very high, approaching 100%, but did not reach statistical significance due to the rarity of these events (**Table 2**).

**Figure 3:**
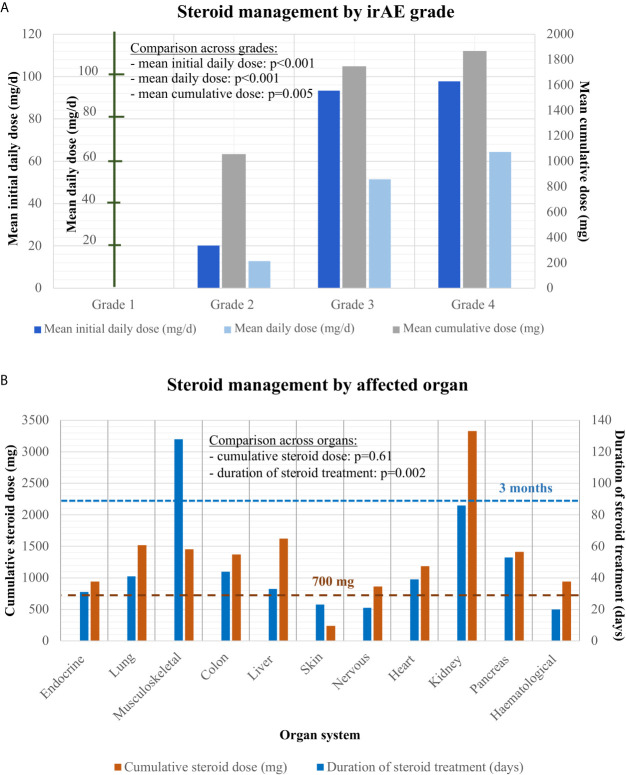
Steroid management of grades 1–4 irAEs. **(A)** Mean initial daily dose, mean average daily dose, and mean cumulative dose for steroid treatment in patients with grade 2–4 irAEs analyzed by one-way ANOVA. While no grade 1 irAE received steroid treatment, mean initial daily, mean average daily and cumulative steroid dose increased steadily from grades 2–4: for grade 2 irAE mean initial dose: 20.2 mg [standard error (SE): 2.9], cumulative dose: 1056.4 mg (SE: 300.3), mean daily dose: 12.9 mg (SE: 2.0); for grade 3 irAE: mean initial dose: 93.4 mg (SE: 9.7), cumulative dose: 1747.5 mg (SE: 186.3), mean daily dose: 51.5 mg (SE: 5.3); for grade 4 irAE mean initial dose: 96.4 mg (SE: 10.9), cumulative dose: 1823.5 mg (SE: 348.4), mean daily dose: 62.8 mg (SE: 8.0). ANOVA with *post-hoc* test for trend across grades: p < 0.001 (mean initial daily dose), p < 0.001 (mean average daily dose), p = 0.005 (cumulative dose). **(B)** Cumulative steroid dose and total duration of steroid treatment by affected organ: endocrine: 942 mg (SE: 536) over 31 days (SE: 16); lungs: 1,519 mg (SE: 248) over 41 days (SE: 6)); musculoskeletal: mean cumulative dose 1455 mg (SE: 442) over 128 days (SE: 33); colon: 1,371 mg (SE: 227) over 44 days (SE: 7); liver: 1,622 mg (SE: 339) over 33 days (SE: 5); skin: 237 mg (SE: 123) over 23 days (SE: 11); nervous system: 863 mg (SE: 427) over 21 days (SE: 9); cardiologic: 1,183 mg (SE: 684) over 39 days (SE: 31); kidney: 3,330 (SE: 2,394) over 86 days (SE: 9); pancreas: 1,413 mg (SE: 1035) over 53 days (SE: 5); blood: 940 mg (SE: na) over 20 days (SE: na). One-way ANOVA p = 0.002 for the cumulative dose across affected organs (with statistical significance in *post-hoc* testing for musculoskeletal irAE, please see **Table 2**), and p = 0.61 for the treatment duration. Abbreviations: irAEs, immune related adverse events; SE, standard error of the mean; n/a, not applicable.

In the majority of cases (66% or 155/232), steroid treatment was required, with increasing frequency and dose for more severe events (**Figure 3A**): no patient with steroid treatment for grade 1 irAE, 51% with grade 2 (mean initial daily dose 20 mg), and >90% with grades 3–4 (mean initial dose 95 mg). The only grade 3–4 cases without steroid therapy were four patients with hypophysitis, who received only hydrocortisone replacement. Overall, hydrocortisone replacement therapy was required for most patients with endocrinologic irAE (24/44 or 55%, namely 22 with hypophysitis, one with thyroiditis, and one with polyendocrinopathy. Utilization of steroid therapy also showed considerable heterogeneity across affected organs and was more frequent for pneumonitis (93%), hepatitis (82%), colitis (81%), and musculoskeletal events (74%, p < 0.001, **Table 2**). Steroid therapy was longest for musculoskeletal irAEs, which were the only type of events with average steroid duration exceeding 3 months (128 days, **Table 2** and **Figure 3B**). Several patients received steroids for >1 year, either higher prednisone doses >10 mg (six patients, all with musculoskeletal irAEs), or low-dose maintenance therapy with ≤10 mg prednisone daily, which was necessary for approximately one-third (14/38 or 37%) of patients with musculoskeletal irAE. On the other hand, the duration of treatment was shortest for skin irAE (average 23 days) and the single case with a hematologic event (20 days, **Table 2**). The cumulative steroid dose was highest for patients with renal (3,330 mg), followed by liver (1,622 mg), and lung irAEs (1,519 mg), but exceeded 700 mg also for all other organs, except for the skin (average 237 mg, **Figure 3B**). A need for additional immunosuppressive therapy was documented in 13/232 events (6%), or 13/198 cases (7%), which corresponded to 13/154 (8.4%) steroid-treated patients: namely 3/28 steroid-treated cases of musculoskeletal events (n = 2 arthritis grade 2, n = 1 arthritis grade 3), 6/30 steroid-treated cases of colitis (n = 1 grade 2, n = 3 grade 3, and n = 2 grade 4), 1/2 steroid-treated cases of myocarditis (grade 4), 2/27 steroid-treated cases of hepatitis (grade 3 and grade 4), and 1/8 steroid-treated cases of dermatitis (grade 3 exacerbated psoriasis). Immunosuppressants administered were mycophenolate mofetil for hepatitis (2×) and myocarditis (1×), mesalamine for colitis (3×), tacrolimus for colitis (1×), infliximab for colitis (2×) and polyarthritis (1×), methotrexate for psoriasis (1×), polyarthritis (1×) and adalimumab as well as leflunomide for arthritis (2× and 1× respectively). Three cases required more than one additional immunosuppressant (n = 2 arthritis, n = 1 colitis). For stage III, steroid therapy was required for most patients with irAEs (79% or 11/14), particularly in case of grade 3–4 events (100% use *vs.* 25% for grade 2, **Supplementary Table 2**).

### Prognostic Impact of irAEs

**Figure 4:**
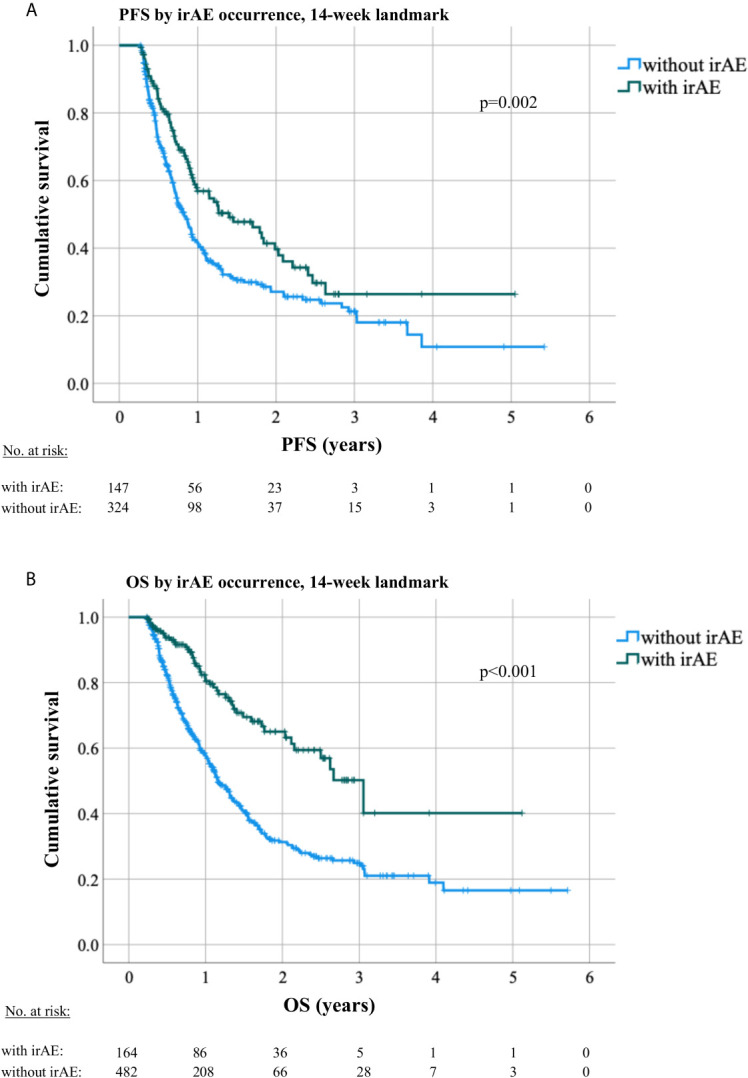
Progression-free and overall survival by occurrence of irAEs in a 14-week landmark analysis. **(A)** The median PFS under immunotherapy was 10 months (8.7–11.4) for patients without irAE *vs.* 17 months (10.3–23.6, logrank p = 0.003) for patients with irAEs in a 14-week landmark analysis. **(B)** The median OS was 15 months (13.5–16.6) for patients without irAE *vs.* 37 months (28.7–44.6, logrank p < 0.001) for patients with irAE in a 14-week landmark analysis.

**Figure 5:**
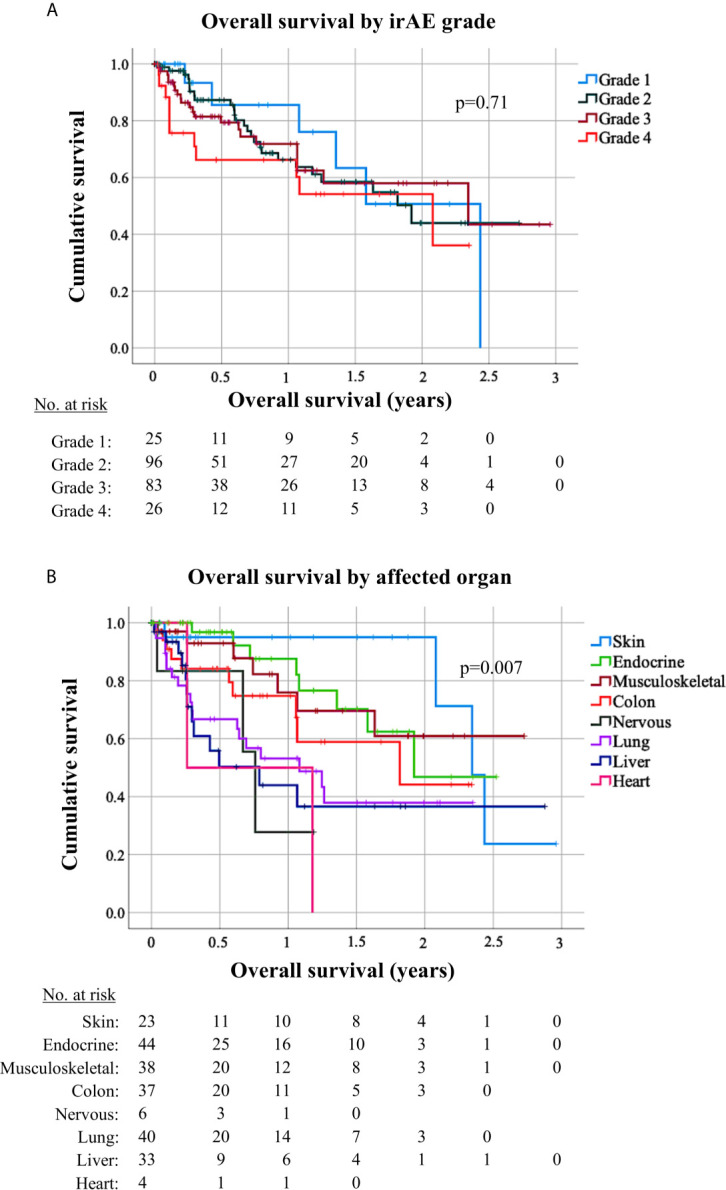
Survival of patients with grade 1–4 immune-related adverse events by affected organ. **(A)** Overall survival (OS) from start of immunotherapy for non-small-cell lung cancer (NSCLC) patients developing immune-related adverse events (irAEs) did not differ significantly by irAE grade (logrank p = 0.71). Median OS was 29 months [95% confidence interval (CI) n/a] in case of grade 1 irAE, 23 months (13.0–31.2) in case of grade 1 irAE, 28 months (3.7–52.6) in case of grade 3 irAE, and 25 months (8.5–41.4) in case of grade 4 irAE. **(B)** OS for NSCLC patients developing irAE showed significant differences according to the irAE type (logrank p = 0.007). Median OS was 28.1 months (CI 23.9–32.3) for patients with dermatologic irAE, with 2-year OS rate 95% (CI 85–100); 23 months (CI n/a) for patients with endocrinologic irAE, with 2-year OS rate 47% (CI 15–79); not reached for patients with musculoskeletal irAE, with a 2-year OS rate 61% (36–85); 22 months (3.1–40.6) for patients with colitis, with a 2-year OS rate 44% (14–75); 13 months (4.2–21.8) for patients with pneumonitis, with a 2-year OS rate 38% (19–57); and 9.5 months (1.4–17.6) for patients with hepatitis, with a 2-year OS rate 37% (14–59); 9.1 months (7.3–10.9) with a 2-year OS rate of 27.8% (CI 0–73) for patients with neurological irAE; and 3.1 months (CI na) for patients with cardiologic irAE with a 2-year OS rate 0%. Only irAE with >3 occurrences in our patients were included in this analysis.

Patients who developed irAEs had a longer PFS [15 *vs*. 9 months, hazard ratio (HR) 0.68 with p = 0.008 in a 12-week landmark analysis, **Supplementary Figure 1A**, **Supplementary Tables 3, 4**; 17 *vs.* 10 months, HR = 0.65 with p = 0.005 in a 14-week landmark analysis, **Figure 4A**, **Tables 3**, **4**], which was significant in multivariable testing along with PD-L1 TPS (p < 0.01), NLR (p > 0.05), treatment line (p > 0.05), type of treatment (chemoimmunotherapy *vs.* IO-monotherapy, p > 0.05), and ECOG PS (p > 0.05, **Table 4** and **Supplementary Table 4**). OS from start of IO treatment was also longer for patients developing irAEs (37 *vs.* 14 months, HR = 0.4 with p < 0.001 in a 12-week landmark analysis, **Supplementary Figure 1B**, **Supplementary Tables 3, 4**; 37 *vs.* 15 months, HR = 0.38 with p < 0.001 in a 14-week landmark analysis, **Figure 4B**, **Tables 3**, **4**), which was significant in multivariable testing along with PD-L1 TPS (p < 0.01), NLR (p < 0.01) treatment line (p > 0.05), type of treatment (p > 0.05), and ECOG PS (p < 0.05, **Table 4**, **Supplementary Table 4**). The independent prognostic value of irAE alongside NLR, PD-L1 TPS, ECOG PS, treatment line, and treatment type was also confirmed in separate multivariable OS analysis using the occurrence of irAE as a time-dependent covariate (**Supplementary Table 5**). OS of patients with irAE varied widely between irAE affecting different organs, being longest for skin (2-year OS 95%), and shortest for pulmonary, hepatic, nervous system, and cardiologic irAE (2-year OS 38, 37, 28, and 0% respectively, p = 0.007, **Figure 5B**) but did not differ significantly by irAE grade (p = 0.71, **Figure 5A**).

**Table 3:** Univariable analysis of progression-free and overall survival according to occurrence of irAE in NSCLC.

PFS with 14-week landmark	HR	P-value	95%-CI
IrAE occurrence	**0.67**	**0.003**	0.51–0.87
PD-L1 TPS (<1, 1–49, 50+)	**0.69**	**<0.001**	0.59–0.82
NLR (≥5, <5)	1.19	0.16	0.93–1.51
Treatment line	1.11	0.14	0.97–1.26
Treatment type^1^	1.30	0.06	1.00–1.70
ECOG PS	1.13	0.31	0.90–1.41
OS with 14-week landmark	HR	P-value	95%-CI
IrAE occurrence	**0.40**	**<0.001**	0.29–0.55
PD-L1 TPS (<1, 1–49, 50+)	**0.77**	**0.002**	0.66–0.91
NLR (≥5, <5)	**1.45**	**0.002**	1.15–1.82
Treatment line	1.18	0.003	1.06–1.32
Treatment type^1^	0.15	0.80	0.59–1.09
ECOG PS	1.22	0.06	0.99–1.52

The association of irAE and other variables with progression-free (PFS) and overall survival (OS) was analyzed using a univariable Cox regression 14-week landmark analysis. Statistically significant results have been highlighted in bold.

PFS, progression-free survival; OS, overall survival; HR, hazard ratio; 95% CI, 95% confidence interval; irAE, immune related adverse events; ECOG PS, Eastern Cooperative Oncology Group Performance Status; IO, immunotherapy; PD-L1 TPS, Programmed Death Ligand 1 Tumor Proportion Score (%); NLR, neutrophil-to-lymphocyte ratio.

^1^chemoimmunotherapy vs. IO-monotherapy.

**Table 4:** Multivariable analysis of progression-free and overall survival according to occurrence of irAE in NSCLC.

PFS with 14-week landmark	HR	P-value	95%-CI
IrAE occurrence	**0.65**	**0.005**	0.48–0.88
PD-L1 TPS (<1, 1–49, 50+)	**0.74**	**0.002**	0.61–0.90
NLR (≥5, <5)	0.99	0.95	0.75–1.29
Treatment line	1.18	0.06	1.00–1.39
Treatment type^1^	1.20	0.36	0.83–1.72
ECOG PS	1.14	0.30	0.89–1.46
**OS with 14-week landmark**	**HR**	**P-value**	**95%-CI**
IrAE occurrence	**0.38**	**<0.001**	0.27–0.56
PD-L1 TPS (<1, 1–49, 50+)	**0.78**	**0.008**	0.66–0.94
NLR (>5, <5)	**1.37**	**0.01**	1.07–1.76
Treatment line	1.15	0.06	0.99–1.32
Treatment type^1^	0.78	0.20	0.54–1.14
ECOG PS	**1.30**	**0.03**	1.02–1.65

The association of irAE and other variables with progression-free (PFS) and overall survival (OS) was analyzed using a multivariable Cox regression 14-week landmark analysis. Statistically significant results have been highlighted in bold.

PFS, progression-free survival; OS, overall survival; HR, hazard ratio; 95% CI, 95% confidence interval; irAE, immune related adverse events; ECOG PS, Eastern Cooperative Oncology Group Performance Status; IO, immunotherapy; PD-L1 TPS, Programmed Death Ligand 1 Tumor Proportion Score (%); NLR, neutrophil-to-lymphocyte ratio.

^1^chemoimmunotherapy vs. IO-monotherapy.

## Discussion

As survival of NSCLC under immunotherapy is improving, with long-term, 5-year OS rates of 20–30% for stage IV disease currently (18, 19), and the use of PD-(L)1 inhibitors is expanding in locally-advanced and early stages (20), the interest for thorough analysis of irAEs is growing, because they pose important practical challenges for oncologists and a major limitation for patient outcome.

The irAE frequency in our study was 22% (189/894) overall, 10.7% (96/894) for grade 3–4 events, and similar between ICI-monotherapy and chemoimmunotherapy, which agrees well with the overall frequency of 20–30% for any grade, and 9–10% for grade 3–4 irAEs reported in the Keynote-24 and Keynote-189 clinical trials (21, 22). The spectrum of involved organs, mainly endocrine glands, lungs, musculoskeletal system, colon, and liver (**Figure 2A**), and median time to onset of 3.1 months were typical and also very similar to that reported by clinical trials and retrospective NSCLC series (21–24). Patient characteristics associated with development of irAEs were PD-L1 positivity (p = 0.003), a lower NLR (p < 0.001), and a better ECOG PS (p = 0.004, **Table 1**). Of note, each of these three parameters is also a predictor of better antitumor efficacy for immunotherapy in NSCLC, both in our patients (**Table 3**) and according to several previous studies (25–29). Therefore, it appears that the efficacy and potential for toxicity are interconnected in case of ICIs. Along the same lines, other studies have linked increase of cytokines, like CXCL9, CXCL10, CXCL11, and CXCL19, under ICIs as a sign of enhanced general immune reactivity with both subsequent tumor responses and development of irAE in NSCLC (30–32). A similar close relationship between efficacy and toxicity is also known to exist in another form of immunotherapy, namely between the graft-*versus*-host and graft-*versus* leukemia effects of allogeneic hematopoietic cell transplantation (33, 34). Besides systemic immunologic parameters, organ-specific factors probably also play a role in the development of specific irAE; for example the frequency of ICI pneumonitis was higher in cases of stage III NSCLC with invariable administration of full-dose thoracic radiotherapy compared to stage IV in our cohort. At the same time, however, it should be noted that palliative radiotherapy was not associated with detectable increase in risk (**Table 1**), which echoes the findings of other investigators and is an important consideration for everyday practice (35, 36). Other examples of organ-related factors that modulate the risk of specific irAE are preexisting interstitial lung disease, which is a strict ICI contraindication due to the very high risk of pneumonitis (37, 38), as well as an increased baseline TSH, which is associated with subsequent development of thyroiditis (39, 40). However, no reliable predictive scheme has been devised yet.

Another clinically important and controversial issue is the relationship between irAE and patient survival (41). Earlier studies in melanoma and NSCLC had shown conflicting results, namely favorable (41–45) or indifferent outcome for patients developing irAEs (46–48), which was in part due to different handling of the “immortal-time bias” (ITB, aka “guaranteed-time bias”) by the various investigators (49). In a recent meta-analysis, both the confounding effect of the ITB and the real, positive association between irAE and patient survival that remains significant after control for ITB could be shown (50). Nevertheless, the relationship between irAE and other predictors for longer PFS and OS, such as PD-L1, NLR, and ECOG PS, evident in our patients (**Table 1**), demonstrates an additional dimension of the question about the potential prognostic utility of irAE, namely whether irAEs have any independent value beyond that of already validated parameters. To our knowledge, the present study is the first to demonstrate this by combining rigorous ITB control using landmark (**Tables 3**, **4**) and time-dependent analyses (**Supplementary Table 5**), with multivariable testing that includes all currently established survival predictors, both laboratory (PD-L1 TPS, NLR) and clinical (treatment type, treatment line, ECOG PS, **Table 4** and **Supplementary Table 5**). Furthermore, particularly relevant for the contemporary practice is the inclusion of a large chemoimmunotherapy subcohort (n > 250, **Table 1**) in this analysis, which is the predominant therapeutic strategy for most NSCLC patients currently (3, 4), in contrast to previous studies who have analyzed IO-monotherapy (41, 50) or small chemoimmunotherapy series with less than 100 patients (51). Our results show that the relationship between occurrence of irAEs and ICI efficacy is very strong (HR = 0.4, **Table 4**), stronger than that of PD-L1 TPS or NLR, and that it persists regardless of concurrent or previous chemotherapy. An additional indication for the potency of this interaction is the lack of negative association between irAE grade and patient survival (**Figure 5A**), which has also been noted by others (52), as well as the recent finding that NSCLC patients with multiple irAEs have an even longer survival (53).

On the other hand, irAEs are increasingly also recognized as a considerable source of patient morbidity and financial burden for the health system (54), with important differences between the real-world and clinical trial setting (55); however systematic studies about routine irAE management are scarce. Of particular interest in this regard are details about the utilization of corticosteroids, which are used much more frequently than other immunosuppressants and have considerable toxicity potential (56). Our results show that the majority or irAEs (67%) will necessitate treatment with steroids, the average cumulative dose of which will exceed 1 g even for grade 2 events (**Figure 3A**). This is important, because cumulative corticosteroid doses >700 mg are known to result in clinically overt impairment of immune function, *i.e.* increased infections (57), which is well in line with the compromised antitumor efficacy of ICIs reported for patients suffering irAEs (58–60). In addition, several other adverse effects, like myopathy, lipodystrophy, memory and mood changes, already commence within the first 1–2 months if the daily dose exceeds 10 mg (61–64), which is the case in the majority of irAEs occurring in NSCLC patients (**Figure 2A**). In contrast, chronic side-effects, like bone density loss, which commences at 3 months (65), and cataracts, the risk of which becomes relevant after 1 year (66), are mainly relevant for patients with musculoskeletal irAEs, of which the average duration of steroid treatment uniquely exceeded 3 months (**Figure 3B**), and about one-third required long-term steroid therapy exceeding 12 months in our study. Indeed, immune checkpoint inhibitor-induced inflammatory arthritis has been described to persist after immunotherapy cessation and necessitates long-term therapy to prevent late relapses, for example with lower-dosed (≤10 mg/day) steroids in combination with disease modifying antirheumatic drugs (67). The multifaceted toxicity of corticosteroids is presumably one main reason, why irAEs that usually present with grade ≥3 and require higher steroid doses, like pneumonitis, colitis and hepatitis (**Table 2**), are associated with shorter OS than irAE affecting other organs (**Figure 5B**). In keeping with this, patients with skin irAEs, who require steroids least frequently (**Table 2**) and have the lowest cumulative dose, uniquely below 700 mg on average (**Figure 3B**), showed the longest OS relative to other irAE types (**Figure 5B**). An association between higher steroid doses and shorter OS in NSCLC patients with irAEs has also been noted by other investigators (68). IrAE fatality in our study was approximately 0.2%, similar to the 0.36–0.38% reported by a global meta-analysis for PD-(L)1 inhibitors across cancer types (69).

The main limitations of our study stem from its retrospective nature, which cannot exclude potential confounders, and is also not as accurate regarding estimation of PFS and other parameters as prospective clinical trials. In order to control this, we annotated our cases extensively and performed multivariable analysis including all factors known to be associated with patient survival. In addition, we accounted for ITB by 12- and 14-week landmark, as well as time-dependent analyses. It should also be noted that our study only enrolled patients with NSCLC from Germany, which limits generalizability of the results to other cancer types and/or other countries with potentially different patterns of clinical practice. Also, for some less frequently affected organs, like the nervous system, heart, and blood, the number of available cases was small and precluded in-depth study.

In conclusion, our study demonstrates that irAEs affect approximately 20–25% of ICI-treated NSCLC patients regardless of additional previous chemotherapy, most necessitating treatment suspension and initiation of steroids. Despite more frequent occurrence with PD-L1 positive tumors, lower NLR, and better ECOG PS, irAEs, particularly those affecting the skin, are independently associated with longer survival. Lethality is below 1%.

## Data Availability Statement

The raw data supporting the conclusions of this article will be made available by the authors without undue reservation.

## Ethics Statement

The studies involving human participants were reviewed and approved by the ethics committee of Heidelberg University (S-296/2016). Written informed consent for participation was not required for this study in accordance with the national legislation and the institutional requirements.

## Author Contributions

LDa: conceptualization, methodology, investigation; data curation, formal analysis, visualization, writing—original draft. ME: investigation, data curation, validation, writing—review & editing. FB: investigation, data curation, validation, writing—review & editing. JK: investigation, data curation, validation, writing—review & editing. DK: investigation; data curation, validation, writing—original draft. HS: investigation, data curation, validation, writing—review & editing. RS: investigation, data curation, validation, writing—review & editing. A-LV: investigation; data curation, validation, writing—review & editing. FL: investigation, data curation, validation, writing—review & editing. LDi: investigation, data curation, validation, writing—review & editing. SL: investigation, data curation, validation, writing—review & editing. MF: investigation, data curation, validation, writing—review & editing. TM: investigation, data curation, validation, writing—review & editing. MK: investigation; data curation, writing—review & editing. BK: validation, supervision, project administration, writing—review & editing. AS: validation, supervision, project administration, writing—review & editing. MT: conceptualization, methodology, validation, supervision, funding acquisition, writing—review & editing. PC: conceptualization, methodology, investigation; data curation, formal analysis, visualization, supervision, project administration; writing—original draft, writing—review & editing. All authors contributed to the article and approved the submitted version.

## Funding

This work was supported by the German Center for Lung Research (DZL).

## Conflict of Interest

FB: research funding from BMS and travel grants from BMS and MSD. JK: research funding from AstraZeneca and Celgene. DK: advisory board and speaker’s honoraria from AstraZeneca, BMS, Pfizer. RS: research funding from BMS. KB: research funding from Novartis and Abbvie, speaker’s honoraria/advisory board/travel grants from Abbvie, BMS, Gilead/Galapagos, Janssen, Lilly, Medac, MSD, Mundipharma, Novartis, Pfizer, Roche, UCB. AS: advisory board honoraria from BMS, AstraZeneca, ThermoFisher, Novartis, speaker’s honoraria from BMS, Illumina, AstraZeneca, Novartis, ThermoFisher, MSD, Roche, and research funding from Chugai. MT: advisory board honoraria from Novartis, Lilly, BMS, MSD, Roche, Celgene, Takeda, AbbVie, Boehringer, speaker’s honoraria from Lilly, MSD, Takeda, research funding from AstraZeneca, BMS, Celgene, Novartis, Roche and travel grants from BMS, MSD, Novartis, Boehringer. PC: research funding from AstraZeneca, Novartis, Roche, Takeda, and advisory board/lecture fees from AstraZeneca, Boehringer Ingelheim, Chugai, Novartis, Pfizer, Roche, Takeda.

The remaining authors declare that the research was conducted in the absence of any commercial or financial relationships that could be construed as a potential conflict of interest.
