# Supplementary material for: Therapeutic and Prognostic Implications of Immune-Related Adverse Events in Advanced Non-Small-Cell Lung Cancer
Source: Front Oncol. 2021 Jun 29;11:703893. doi: 10.3389/fonc.2021.703893 (PMC8277237; doi:10.3389/fonc.2021.703893)
Supplement: Supplementary file 1 [file DataSheet_1.pdf]

Supplementary Material

Supplementary Table 1: Characteristics of stage III non-small cell lung cancer patients who received durvalumab after definitive chemoradiation

**All Patients (n=45)**	**Patients without irAE (N=31)**	**Patients with irAE (N=14)**	**p-value**
**Age, median**	66.9	67.4	0.78
**Sex, male/female**	13/13	12/2	0.03
**never/light-smokers (<10 py)**	0	0	
**pack-years, mean**	41.5	47.5	0.23
**ECOG PS 0 / 1, number**	13 / 11	9 / 5	0.5
**PD-L1 TPS, mean**	38.1	33.4	0.95
**NLR, mean**	7.0	5.6	0.45

Abbreviations: irAE: immune-related adverse events; ECOG PS: ECOG performance status; NLR: neutrophil-to-lymphocyte ratio.

Supplementary Table 2: Description of irAEs, their severity and management

**Affected organs**	**Patients (N)**	**Grade ≥3 (N)**	**Steroids (N)**
Pneumonitis	7	6	7
Dermatitis	1	0	0
Endocrinological	4	1	2
Hepatitis	2	1	2

Supplementary Table 3: Univariable 12-week landmark analysis of progression-free and overall survival according to occurrence of irAE in NSCLC

The association of irAE and other variables with progression-free (PFS) and overall survival (OS) was analyzed using a univariable Cox regression 12- landmark analysis. Statistically significant results have been highlighted in bold.

**PFS with 12-week landmark**	**HR**	**P-value**	**95%-CI**
IrAE occurrence	**0.68**	**0.004**	0.53-0.88
PD-L1 TPS (<1, 1-49, 50+)	**0.70**	**<0.001**	0.59-0.82
NLR (≥5, <5)	1.20	1.20	0.95-1.51
Treatment line	1.11	0.09	0.98-1.26
Treatment type [1]	1.16	0.26	0.90-1.51
ECOG PS	1.13	0.29	0.91-1.40
**OS with 12-week landmark**	**HR**	**P-value**	**95%-CI**
IrAE occurrence	**0.40**	**<0.001**	0.29-0.55
PD-L1 TPS (<1, 1-49, 50+)	**0.77**	**0.001**	0.66-0.89
NLR (≥5, <5)	1.52	<0.001	1.21-1.90
Treatment line	**1.19**	**0.001**	1.07-1.33
Treatment type [1]	0.75	0.06	0.56-1.01
ECOG PS	**1.26**	**0.03**	1.03-1.55

Abbreviations: PFS: progression-free survival; OS: overall survival; HR: hazard ratio; 95% CI: 95% confidence interval; irAE: immune related adverse events; ECOG PS: Eastern Cooperative Oncology Group Performance Status; IO: immunotherapy; PD-L1 TPS: Programmed Death Ligand 1 Tumor Proportion Score (%); NLR: Neutrophil-to-Lymphocyte Ratio.

[1] chemoimmunotherapy *vs.* IO- monotherapy.

Supplementary Table 4: Multivariable 12-week landmark analysis of progression-free and overall survival according to occurrence of irAE in NSCLC

The association of irAE and other variables with progression-free (PFS) and overall survival (OS) was analyzed using a multivariable Cox regression 12-week landmark analysis. Statistically significant results have been highlighted in bold.

**PFS with 12-week landmark**	**HR**	**P-value**	**95%-CI**
IrAE occurrence	**0.68**	**0.009**	0.51-0.90
PD-L1 TPS (<1, 1-49, 50+)	**0.71**	**<0.001**	0.59-0.86
NLR (≥5, <5)	1.05	0.73	0.80-1.34
Treatment line	1.14	0.12	0.97-1.33
Treatment type [1]	0.98	0.91	0.70-1.40
ECOG PS	1.16	0.23	0.91-1.45
**OS with 12-week landmark**	**HR**	**P-value**	**95%-CI**
IrAE occurrence	**0.40**	**<0.001**	0.28-0.58
PD-L1 TPS (<1, 1-49, 50+)	**0.76**	**0.003**	0.64-0.91
NLR (≥5, <5)	**1.45**	**0.003**	1.13-1.86
Treatment line	1.13	0.08	0.99-1.30
Treatment type [1]	0.71	0.07	0.49-1.03
ECOG PS	**1.35**	**0.01**	1.07-1.71

<u>Abbreviations:</u> PFS: progression-free survival; OS: overall survival; HR: hazard ratio; 95% CI: 95% confidence interval; irAE: immune related adverse events; ECOG PS: Eastern Cooperative Oncology Group Performance Status; IO: immunotherapy; PD-L1 TPS: Programmed Death Ligand 1 Tumor Proportion Score (%); NLR: Neutrophil-to-Lymphocyte Ratio.

[1] chemoimmunotherapy vs. IO- monotherapy.

Supplementary Table 5: Multivariable time-dependent Cox regression analysis of overall survival

The association of irAE and other variables with overall survival (OS) was analyzed using a multivariable Cox regression with immune-related adverse events (irAE) as a time dependent co-variate. Included were all parameters used in the multivariable analysis of Table 4. Statistically significant results have been highlighted.

[1] OS, multivariable analysis	HR	P-value	95% CI
**irAE**	**0.70**	**0.03**	**0.51-0.97**
**PD-L1 TPS (<1, 1-49, 50+)**	**0.77**	**<0.001**	**0.66-0.89**
**NLR (≥5, <5)**	**1.91**	**<0.001**	**1.55-2.37**
**treatment line**	**1.14**	**0.01**	**1.03-1.27**
**treatment type [1]**	**0.47**	**<0.001**	**0.34-0.64**
**ECOG PS**	**1.45**	**<0.001**	**1.20-1.75**

Abbreviations: PFS, progression-free survival; OS, overall survival, HR, hazard ratio; 95% CI: 95% confidence interval; irAE, immune-related adverse events; ECOG PS, Eastern Cooperative Oncology Group performance status; IO, immunotherapy; PD-L1 TPS, Programmed Death Ligand 1 Tumor Proportion Score (%); NLR: Neutrophil-to-Lymphocyte Ratio.

[1] chemoimmunotherapy *vs.* IO- monotherapy

Supplementary Figure 1. Progression-free and overall survival by occurrence of irAE in a 12-week landmark analysis

(A) The median progression-free survival under immunotherapy (PFS) was 9 months (95% confidence interval [CI] 8.1-10.4) for patients without immune-related adverse events (irAE) *vs.* 15 months (8.2-22.1, logrank p=0.003) for patients with irAE in a 12-week landmark analysis.

(B) The median overall survival from immunotherapy start (OS) was 14 months (12.4-15.6) for patients without irAE *vs.* 37 months (28.5-44.8, logrank p<0.001) for patients with irAE in a 12-week landmark analysis.

Supplementary Figure 1

A.

PFS by irAE occurrence, 12-week landmark

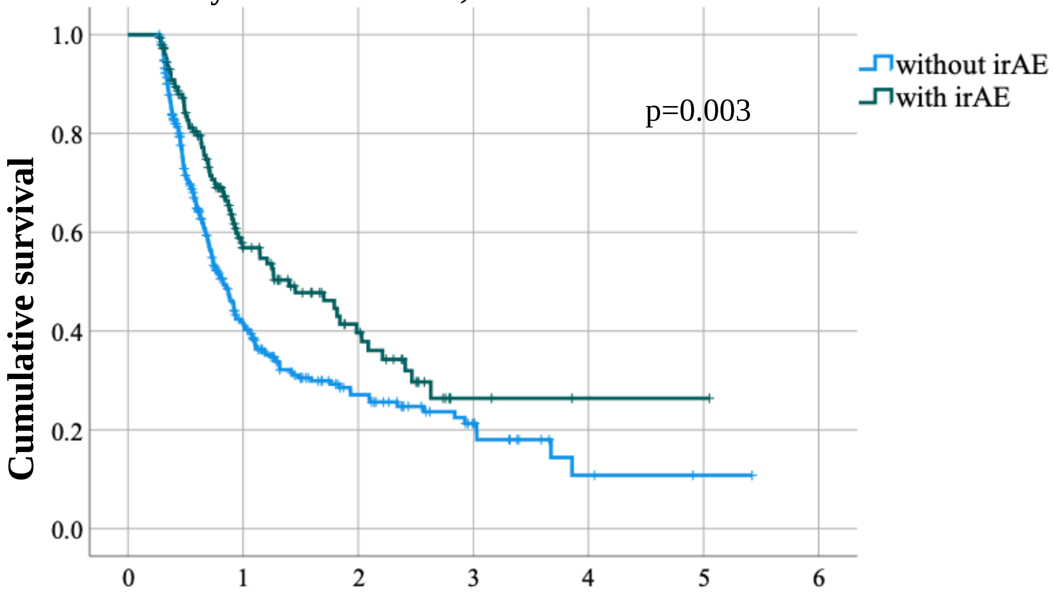

PFS (years)

No. at risk:

with irAE:	156	56	23	3	1	1	0
without irAE:	350	98	37	15	3	1	0

B.

OS by irAE occurrence, 12-week landmark

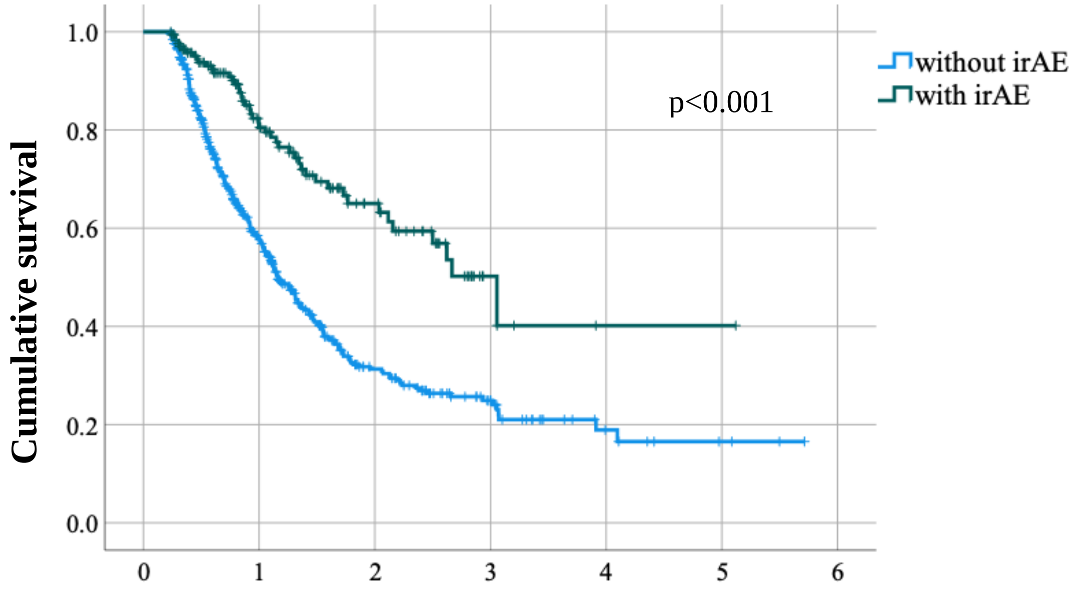

OS (years)

No. at risk:

with irAE:	171	86	36	5	1	1	0
without irAE:	503	208	66	28	7	3	0
